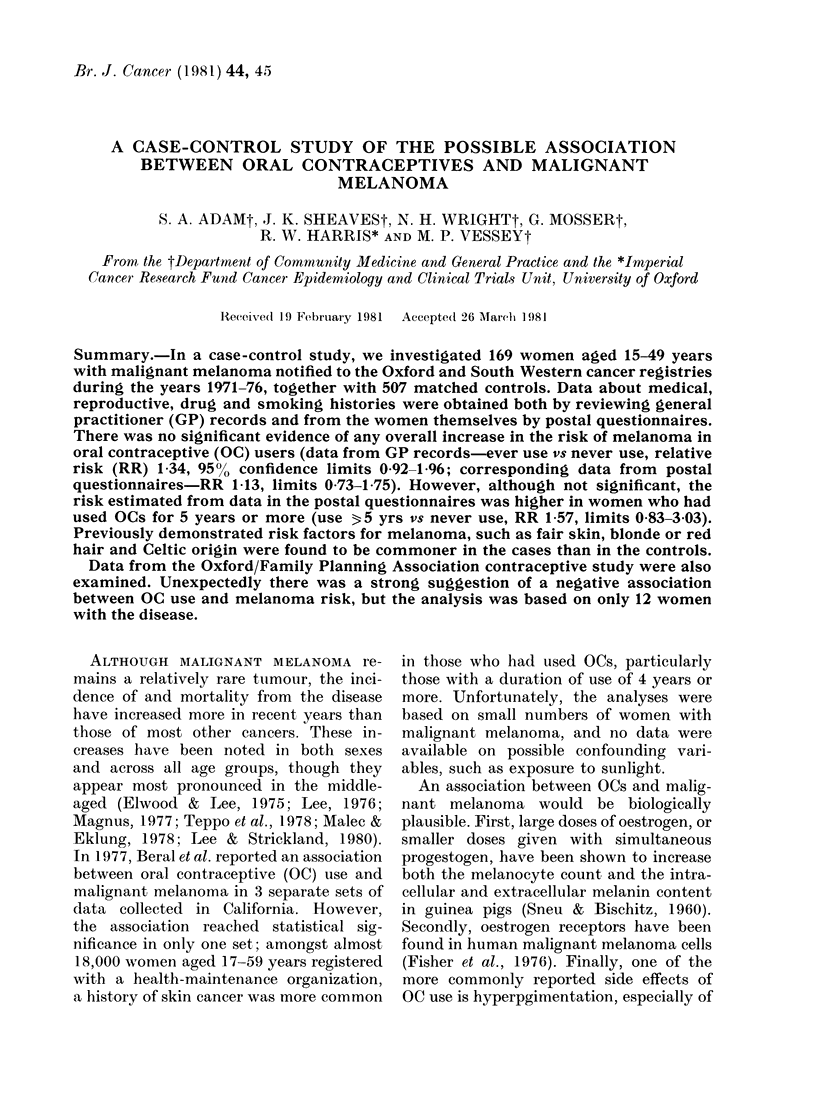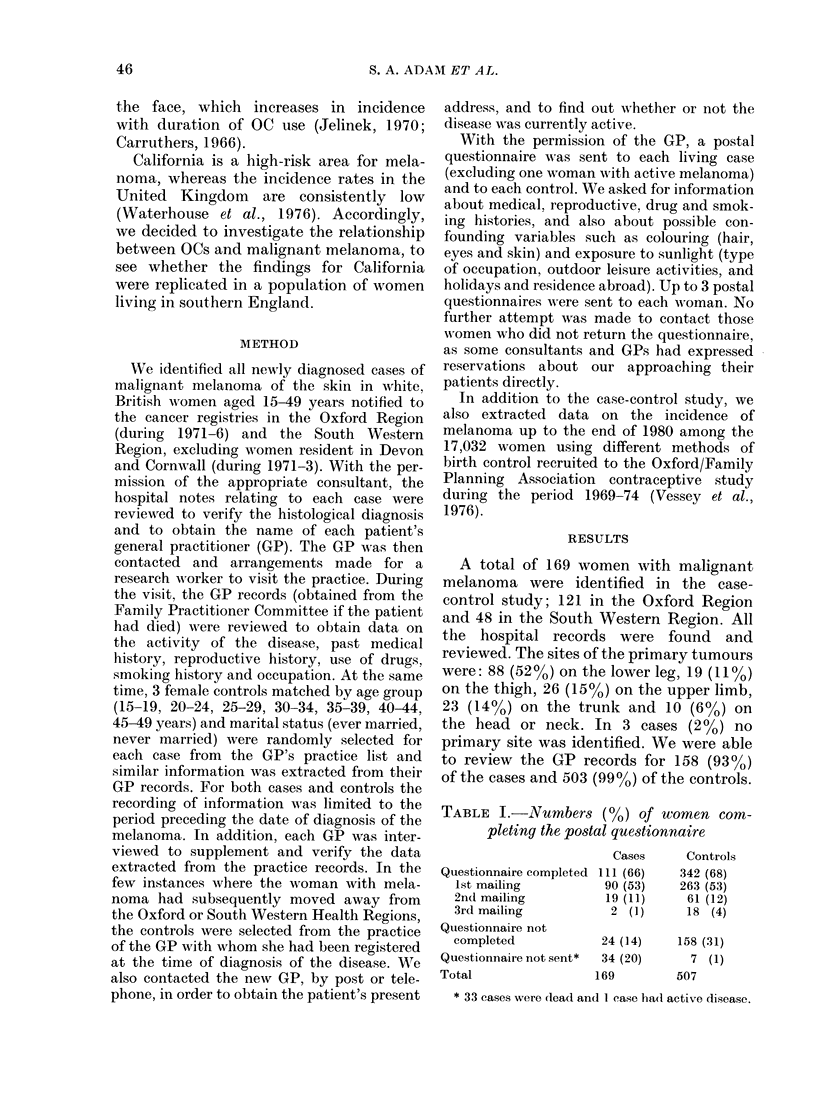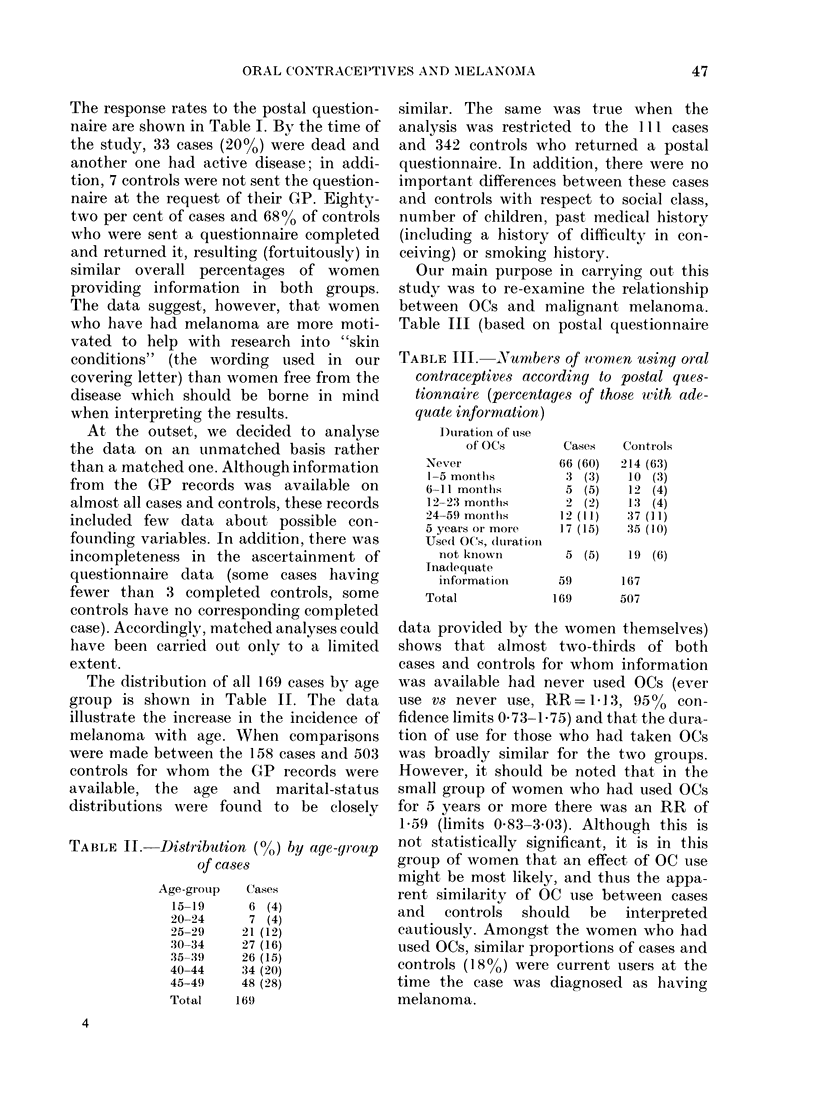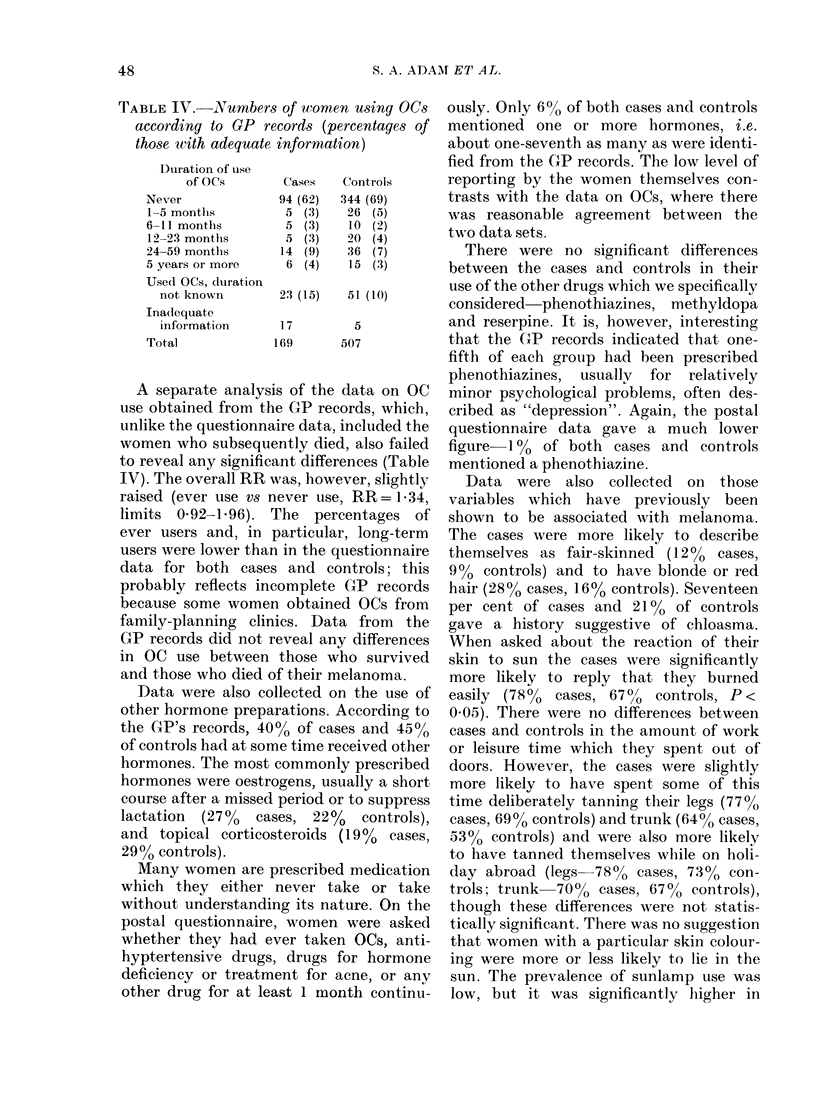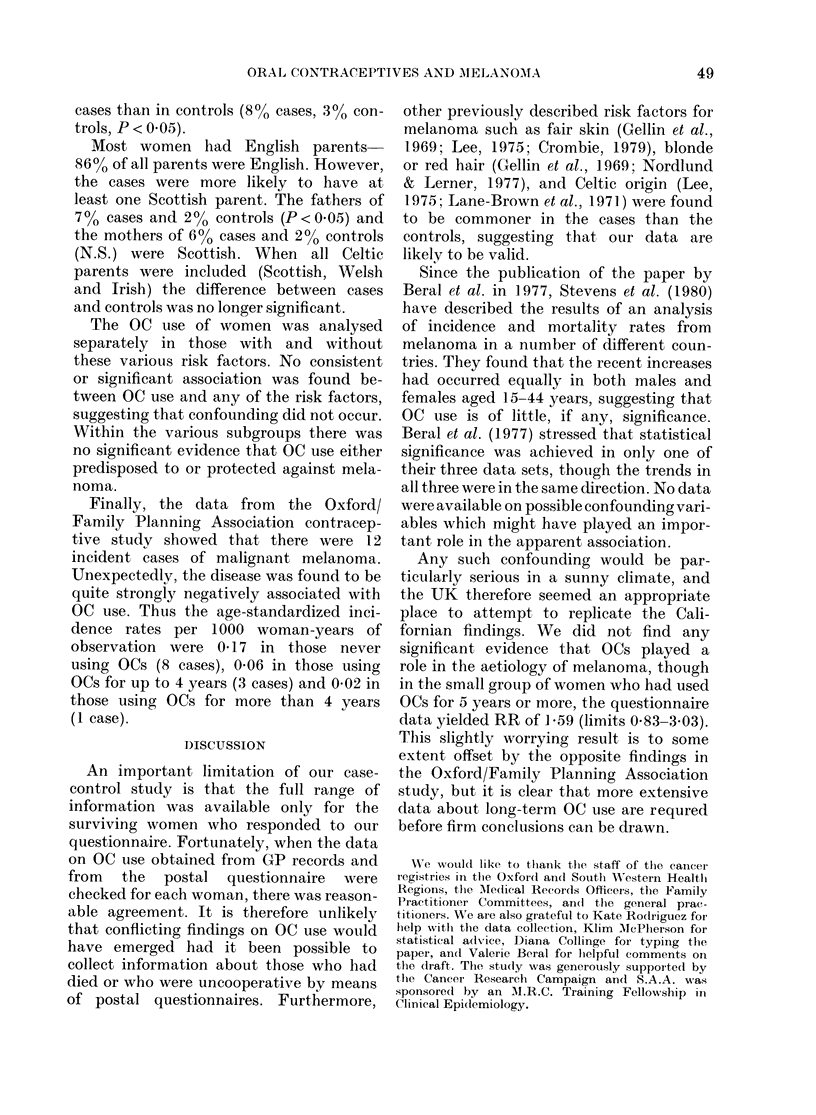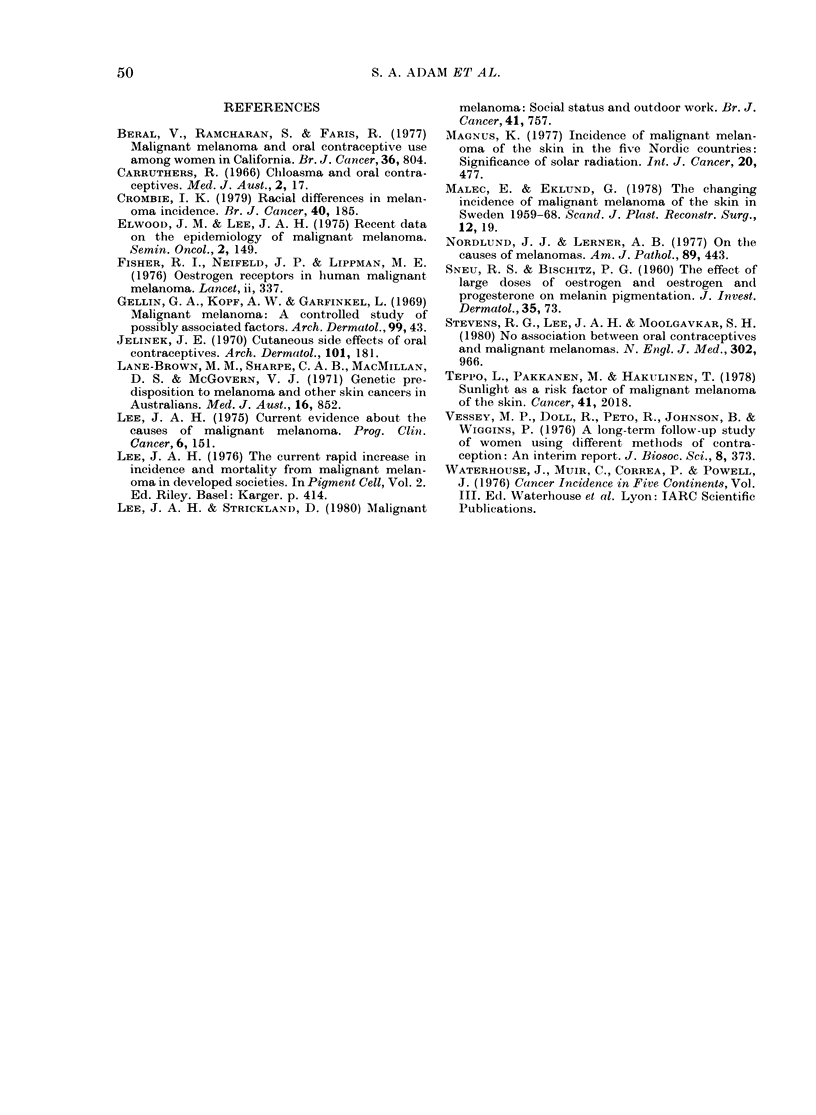# A case-control study of the possible association between oral contraceptives and malignant melanoma.

**DOI:** 10.1038/bjc.1981.145

**Published:** 1981-07

**Authors:** S. A. Adam, J. K. Sheaves, N. H. Wright, G. Mosser, R. W. Harris, M. P. Vessey

## Abstract

In a case-control study, we investigated 169 women aged 15-49 years with malignant melanoma notified to the Oxford and South Western cancer registries during the years 1971-1976, together with 507 matched controls. Data about medical, reproductive, drug and smoking histories were obtained both by reviewing general practitioner (GP) records and from the women themselves by postal questionnaires. There was no significant evidence of any overall increase in the risk of melanoma in oral contraceptive (OC) users (data from GP records-ever use vs never use, relative risk (RR) 1.34, 95% confidence limits 0.92-1.96; corresponding data from postal questionnaires-RR 1.13, limits 0.73-1.75). However, although not significant, the risk estimated from data in the postal questionnaires was higher in women who had used OCs for 5 years or more (use greater than or equal to 5 years vs never use, RR 1.57, limits 0.83-3.03). Previously demonstrated risk factors for melanoma, such as fair skin, blond or red hair and Celtic origin were found to be commoner in the cases than in the controls. Data from the Oxford/Family Planning Association contraceptive study were also examined. Unexpectedly there was a strong suggestion of a negative association between OC use and melanoma risk, but the analysis was based on only 12 women with the disease.


*Br. J. Cancer* (1981) **44**, 45

### A CASE-CONTROL STUDY OF THE POSSIBLE ASSOCIATION BETWEEN ORAL CONTRACEPTIVES AND MALIGNANT MELANOMA

S. A. ADAM†, J. K. SHEAVES†, N. H. WRIGHT†, G. MOSSER†, R. W. HARRIS* AND M. P. VESSEY†

*From the †Department of Community Medicine and General Practice and the *Imperial Cancer Research Fund Cancer Epidemiology and Clinical Trials Unit, University of Oxford*

Received 19 February 1981 Accepted 26 March 1981

**Summary.—In a case-control study, we investigated 169 women aged 15–49 years with malignant melanoma notified to the Oxford and South Western cancer registries during the years 1971–76, together with 507 matched controls. Data about medical, reproductive, drug and smoking histories were obtained both by reviewing general practitioner (GP) records and from the women themselves by postal questionnaires. There was no significant evidence of any overall increase in the risk of melanoma in oral contraceptive (OC) users (data from GP records—ever use *vs* never use, relative risk (RR) 1·34, 95% confidence limits 0·92–1·96; corresponding data from postal questionnaires—RR 1·13, limits 0·73–1·75). However, although not significant, the risk estimated from data in the postal questionnaires was higher in women who had used OCs for 5 years or more (use $\geqslant$5 yrs *vs* never use, RR 1·57, limits 0·83–3·03). Previously demonstrated risk factors for melanoma, such as fair skin, blonde or red hair and Celtic origin were found to be commoner in the cases than in the controls.**

**Data from the Oxford/Family Planning Association contraceptive study were also examined. Unexpectedly there was a strong suggestion of a negative association between OC use and melanoma risk, but the analysis was based on only 12 women with the disease.**

ALTHOUGH MALIGNANT MELANOMA remains a relatively rare tumour, the incidence of and mortality from the disease have increased more in recent years than those of most other cancers. These increases have been noted in both sexes and across all age groups, though they appear most pronounced in the middle-aged (Elwood & Lee, 1975; Lee, 1976; Magnus, 1977; Teppo *et al.*, 1978; Malec & Eklung, 1978; Lee & Strickland, 1980). In 1977, Beral *et al.* reported an association between oral contraceptive (OC) use and malignant melanoma in 3 separate sets of data collected in California. However, the association reached statistical significance in only one set; amongst almost 18,000 women aged 17–59 years registered with a health-maintenance organization, a history of skin cancer was more common in those who had used OCs, particularly those with a duration of use of 4 years or more. Unfortunately, the analyses were based on small numbers of women with malignant melanoma, and no data were available on possible confounding variables, such as exposure to sunlight.

An association between OCs and malignant melanoma would be biologically plausible. First, large doses of oestrogen, or smaller doses given with simultaneous progestogen, have been shown to increase both the melanocyte count and the intracellular and extracellular melanin content in guinea pigs (Sneu & Bischitz, 1960). Secondly, oestrogen receptors have been found in human malignant melanoma cells (Fisher *et al.*, 1976). Finally, one of the more commonly reported side effects of OC use is hyperpgimentation, especially of the face, which increases in incidence with duration of OC use (Jelinek, 1970; Carruthers, 1966).

California is a high-risk area for melanoma, whereas the incidence rates in the United Kingdom are consistently low (Waterhouse *et al.*, 1976). Accordingly, we decided to investigate the relationship between OCs and malignant melanoma, to see whether the findings for California were replicated in a population of women living in southern England.

### METHOD

We identified all newly diagnosed cases of malignant melanoma of the skin in white, British women aged 15–49 years notified to the cancer registries in the Oxford Region (during 1971–6) and the South Western Region, excluding women resident in Devon and Cornwall (during 1971–3). With the permission of the appropriate consultant, the hospital notes relating to each case were reviewed to verify the histological diagnosis and to obtain the name of each patient's general practitioner (GP). The GP was then contacted and arrangements made for a research worker to visit the practice. During the visit, the GP records (obtained from the Family Practitioner Committee if the patient had died) were reviewed to obtain data on the activity of the disease, past medical history, reproductive history, use of drugs, smoking history and occupation. At the same time, 3 female controls matched by age group (15–19, 20–24, 25–29, 30–34, 35–39, 40–44, 45–49 years) and marital status (ever married, never married) were randomly selected for each case from the GP's practice list and similar information was extracted from their GP records. For both cases and controls the recording of information was limited to the period preceding the date of diagnosis of the melanoma. In addition, each GP was interviewed to supplement and verify the data extracted from the practice records. In the few instances where the woman with melanoma had subsequently moved away from the Oxford or South Western Health Regions, the controls were selected from the practice of the GP with whom she had been registered at the time of diagnosis of the disease. We also contacted the new GP, by post or telephone, in order to obtain the patient's present address, and to find out whether or not the disease was currently active.

With the permission of the GP, a postal questionnaire was sent to each living case (excluding one woman with active melanoma) and to each control. We asked for information about medical, reproductive, drug and smoking histories, and also about possible confounding variables such as colouring (hair, eyes and skin) and exposure to sunlight (type of occupation, outdoor leisure activities, and holidays and residence abroad). Up to 3 postal questionnaires were sent to each woman. No further attempt was made to contact those women who did not return the questionnaire, as some consultants and GPs had expressed reservations about our approaching their patients directly.

In addition to the case-control study, we also extracted data on the incidence of melanoma up to the end of 1980 among the 17,032 women using different methods of birth control recruited to the Oxford/Family Planning Association contraceptive study during the period 1969–74 (Vessey *et al.*, 1976).

### RESULTS

A total of 169 women with malignant melanoma were identified in the case-control study; 121 in the Oxford Region and 48 in the South Western Region. All the hospital records were found and reviewed. The sites of the primary tumours were: 88 (52%) on the lower leg, 19 (11%) on the thigh, 26 (15%) on the upper limb, 23 (14%) on the trunk and 10 (6%) on the head or neck. In 3 cases (2%) no primary site was identified. We were able to review the GP records for 158 (93%) of the cases and 503 (99%) of the controls.

TABLE I.—*Numbers (%) of women completing the postal questionnaire*

| | Cases | Controls |
|---|---|---|
| Questionnaire completed | 111 (66) | 342 (68) |
| 1st mailing | 90 (53) | 263 (53) |
| 2nd mailing | 19 (11) | 61 (12) |
| 3rd mailing | 2 (1) | 18 (4) |
| Questionnaire not completed | 24 (14) | 158 (31) |
| Questionnaire not sent* | 34 (20) | 7 (1) |
| Total | 169 | 507 |

* 33 cases were dead and 1 case had active disease.

The response rates to the postal questionnaire are shown in Table I. By the time of the study, 33 cases (20%) were dead and another one had active disease; in addition, 7 controls were not sent the questionnaire at the request of their GP. Eighty-two per cent of cases and 68% of controls who were sent a questionnaire completed and returned it, resulting (fortuitously) in similar overall percentages of women providing information in both groups. The data suggest, however, that women who have had melanoma are more motivated to help with research into "skin conditions" (the wording used in our covering letter) than women free from the disease which should be borne in mind when interpreting the results.

At the outset, we decided to analyse the data on an unmatched basis rather than a matched one. Although information from the GP records was available on almost all cases and controls, these records included few data about possible confounding variables. In addition, there was incompleteness in the ascertainment of questionnaire data (some cases having fewer than 3 completed controls, some controls have no corresponding completed case). Accordingly, matched analyses could have been carried out only to a limited extent.

The distribution of all 169 cases by age group is shown in Table II. The data illustrate the increase in the incidence of melanoma with age. When comparisons were made between the 158 cases and 503 controls for whom the GP records were available, the age and marital-status distributions were found to be closely similar. The same was true when the analysis was restricted to the 111 cases and 342 controls who returned a postal questionnaire. In addition, there were no important differences between these cases and controls with respect to social class, number of children, past medical history (including a history of difficulty in conceiving) or smoking history.

TABLE II.—*Distribution (%) by age-group of cases*

| Age-group | Cases |
|---|---|
| 15–19 | 6 (4) |
| 20–24 | 7 (4) |
| 25–29 | 21 (12) |
| 30–34 | 27 (16) |
| 35–39 | 26 (15) |
| 40–44 | 34 (20) |
| 45–49 | 48 (28) |
| Total | 169 |

Our main purpose in carrying out this study was to re-examine the relationship between OCs and malignant melanoma. Table III (based on postal questionnaire data provided by the women themselves) shows that almost two-thirds of both cases and controls for whom information was available had never used OCs (ever use *vs* never use, RR = 1·13, 95% confidence limits 0·73–1·75) and that the duration of use for those who had taken OCs was broadly similar for the two groups. However, it should be noted that in the small group of women who had used OCs for 5 years or more there was an RR of 1·59 (limits 0·83–3·03). Although this is not statistically significant, it is in this group of women that an effect of OC use might be most likely, and thus the apparent similarity of OC use between cases and controls should be interpreted cautiously. Amongst the women who had used OCs, similar proportions of cases and controls (18%) were current users at the time the case was diagnosed as having melanoma.

TABLE III.—*Numbers of women using oral contraceptives according to postal questionnaire (percentages of those with adequate information)*

| Duration of use of OCs | Cases | Controls |
|---|---|---|
| Never | 66 (60) | 214 (63) |
| 1–5 months | 3 (3) | 10 (3) |
| 6–11 months | 5 (5) | 12 (4) |
| 12–23 months | 2 (2) | 13 (4) |
| 24–59 months | 12 (11) | 37 (11) |
| 5 years or more | 17 (15) | 35 (10) |
| Used OCs, duration not known | 5 (5) | 19 (6) |
| Inadequate information | 59 | 167 |
| Total | 169 | 507 |

TABLE IV.—*Numbers of women using OCs according to GP records (percentages of those with adequate information)*

| Duration of use of OCs | Cases | Controls |
|---|---|---|
| Never | 94 (62) | 344 (69) |
| 1–5 months | 5 (3) | 26 (5) |
| 6–11 months | 5 (3) | 10 (2) |
| 12–23 months | 5 (3) | 20 (4) |
| 24–59 months | 14 (9) | 36 (7) |
| 5 years or more | 6 (4) | 15 (3) |
| Used OCs, duration not known | 23 (15) | 51 (10) |
| Inadequate information | 17 | 5 |
| Total | 169 | 507 |

A separate analysis of the data on OC use obtained from the GP records, which, unlike the questionnaire data, included the women who subsequently died, also failed to reveal any significant differences (Table IV). The overall RR was, however, slightly raised (ever use *vs* never use, RR = 1·34, limits 0·92–1·96). The percentages of ever users and, in particular, long-term users were lower than in the questionnaire data for both cases and controls; this probably reflects incomplete GP records because some women obtained OCs from family-planning clinics. Data from the GP records did not reveal any differences in OC use between those who survived and those who died of their melanoma.

Data were also collected on the use of other hormone preparations. According to the GP's records, 40% of cases and 45% of controls had at some time received other hormones. The most commonly prescribed hormones were oestrogens, usually a short course after a missed period or to suppress lactation (27% cases, 22% controls), and topical corticosteroids (19% cases, 29% controls).

Many women are prescribed medication which they either never take or take without understanding its nature. On the postal questionnaire, women were asked whether they had ever taken OCs, anti-hyptertensive drugs, drugs for hormone deficiency or treatment for acne, or any other drug for at least 1 month continuously. Only 6% of both cases and controls mentioned one or more hormones, *i.e.* about one-seventh as many as were identified from the GP records. The low level of reporting by the women themselves contrasts with the data on OCs, where there was reasonable agreement between the two data sets.

There were no significant differences between the cases and controls in their use of the other drugs which we specifically considered—phenothiazines, methyldopa and reserpine. It is, however, interesting that the GP records indicated that one-fifth of each group had been prescribed phenothiazines, usually for relatively minor psychological problems, often described as "depression". Again, the postal questionnaire data gave a much lower figure—1% of both cases and controls mentioned a phenothiazine.

Data were also collected on those variables which have previously been shown to be associated with melanoma. The cases were more likely to describe themselves as fair-skinned (12% cases, 9% controls) and to have blonde or red hair (28% cases, 16% controls). Seventeen per cent of cases and 21% of controls gave a history suggestive of chloasma. When asked about the reaction of their skin to sun the cases were significantly more likely to reply that they burned easily (78% cases, 67% controls, $P < 0{\cdot}05$). There were no differences between cases and controls in the amount of work or leisure time which they spent out of doors. However, the cases were slightly more likely to have spent some of this time deliberately tanning their legs (77% cases, 69% controls) and trunk (64% cases, 53% controls) and were also more likely to have tanned themselves while on holiday abroad (legs—78% cases, 73% controls; trunk—70% cases, 67% controls), though these differences were not statistically significant. There was no suggestion that women with a particular skin colouring were more or less likely to lie in the sun. The prevalence of sunlamp use was low, but it was significantly higher in cases than in controls (8% cases, 3% controls, $P < 0{\cdot}05$).

Most women had English parents—86% of all parents were English. However, the cases were more likely to have at least one Scottish parent. The fathers of 7% cases and 2% controls ($P < 0{\cdot}05$) and the mothers of 6% cases and 2% controls (N.S.) were Scottish. When all Celtic parents were included (Scottish, Welsh and Irish) the difference between cases and controls was no longer significant.

The OC use of women was analysed separately in those with and without these various risk factors. No consistent or significant association was found between OC use and any of the risk factors, suggesting that confounding did not occur. Within the various subgroups there was no significant evidence that OC use either predisposed to or protected against melanoma.

Finally, the data from the Oxford/Family Planning Association contraceptive study showed that there were 12 incident cases of malignant melanoma. Unexpectedly, the disease was found to be quite strongly negatively associated with OC use. Thus the age-standardized incidence rates per 1000 woman-years of observation were 0·17 in those never using OCs (8 cases), 0·06 in those using OCs for up to 4 years (3 cases) and 0·02 in those using OCs for more than 4 years (1 case).

### DISCUSSION

An important limitation of our case-control study is that the full range of information was available only for the surviving women who responded to our questionnaire. Fortunately, when the data on OC use obtained from GP records and from the postal questionnaire were checked for each woman, there was reasonable agreement. It is therefore unlikely that conflicting findings on OC use would have emerged had it been possible to collect information about those who had died or who were uncooperative by means of postal questionnaires. Furthermore, other previously described risk factors for melanoma such as fair skin (Gellin *et al.*, 1969; Lee, 1975; Crombie, 1979), blonde or red hair (Gellin *et al.*, 1969; Nordlund & Lerner, 1977), and Celtic origin (Lee, 1975; Lane-Brown *et al.*, 1971) were found to be commoner in the cases than the controls, suggesting that our data are likely to be valid.

Since the publication of the paper by Beral *et al.* in 1977, Stevens *et al.* (1980) have described the results of an analysis of incidence and mortality rates from melanoma in a number of different countries. They found that the recent increases had occurred equally in both males and females aged 15–44 years, suggesting that OC use is of little, if any, significance. Beral *et al.* (1977) stressed that statistical significance was achieved in only one of their three data sets, though the trends in all three were in the same direction. No data were available on possible confounding variables which might have played an important role in the apparent association.

Any such confounding would be particularly serious in a sunny climate, and the UK therefore seemed an appropriate place to attempt to replicate the Californian findings. We did not find any significant evidence that OCs played a role in the aetiology of melanoma, though in the small group of women who had used OCs for 5 years or more, the questionnaire data yielded RR of 1·59 (limits 0·83–3·03). This slightly worrying result is to some extent offset by the opposite findings in the Oxford/Family Planning Association study, but it is clear that more extensive data about long-term OC use are requred before firm conclusions can be drawn.

We would like to thank the staff of the cancer registries in the Oxford and South Western Health Regions, the Medical Records Officers, the Family Practitioner Committees, and the general practitioners. We are also grateful to Kate Rodriguez for help with the data collection, Klim McPherson for statistical advice, Diana Collinge for typing the paper, and Valerie Beral for helpful comments on the draft. The study was generously supported by the Cancer Research Campaign and S.A.A. was sponsored by an M.R.C. Training Fellowship in Clinical Epidemiology.

### REFERENCES

BERAL, V., RAMCHARAN, S. & FARIS, R. (1977) Malignant melanoma and oral contraceptive use among women in California. *Br. J. Cancer*, **36**, 804.

CARRUTHERS, R. (1966) Chloasma and oral contraceptives. *Med. J. Aust.*, **2**, 17.

CROMBIE, I. K. (1979) Racial differences in melanoma incidence. *Br. J. Cancer*, **40**, 185.

ELWOOD, J. M. & LEE, J. A. H. (1975) Recent data on the epidemiology of malignant melanoma. *Semin. Oncol.*, **2**, 149.

FISHER, R. I., NEIFELD, J. P. & LIPPMAN, M. E. (1976) Oestrogen receptors in human malignant melanoma. *Lancet*, ii, 337.

GELLIN, G. A., KOPF, A. W. & GARFINKEL, L. (1969) Malignant melanoma: A controlled study of possibly associated factors. *Arch. Dermatol.*, **99**, 43.

JELINEK, J. E. (1970) Cutaneous side effects of oral contraceptives. *Arch. Dermatol.*, **101**, 181.

LANE-BROWN, M. M., SHARPE, C. A. B., MACMILLAN, D. S. & MCGOVERN, V. J. (1971) Genetic predisposition to melanoma and other skin cancers in Australians. *Med. J. Aust.*, **16**, 852.

LEE, J. A. H. (1975) Current evidence about the causes of malignant melanoma. *Prog. Clin. Cancer*, **6**, 151.

LEE, J. A. H. (1976) The current rapid increase in incidence and mortality from malignant melanoma in developed societies. In *Pigment Cell*, Vol. 2. Ed. Riley. Basel: Karger. p. 414.

LEE, J. A. H. & STRICKLAND, D. (1980) Malignant melanoma: Social status and outdoor work. *Br. J. Cancer*, **41**, 757.

MAGNUS, K. (1977) Incidence of malignant melanoma of the skin in the five Nordic countries: Significance of solar radiation. *Int. J. Cancer*, **20**, 477.

MALEC, E. & EKLUND, G. (1978) The changing incidence of malignant melanoma of the skin in Sweden 1959-68. *Scand. J. Plast. Reconstr. Surg.*, **12**, 19.

NORDLUND, J. J. & LERNER, A. B. (1977) On the causes of melanomas. *Am. J. Pathol.*, **89**, 443.

SNEU, R. S. & BISCHITZ, P. G. (1960) The effect of large doses of oestrogen and oestrogen and progesterone on melanin pigmentation. *J. Invest. Dermatol.*, **35**, 73.

STEVENS, R. G., LEE, J. A. H. & MOOLGAVKAR, S. H. (1980) No association between oral contraceptives and malignant melanomas. *N. Engl. J. Med.*, **302**, 966.

TEPPO, L., PAKKANEN, M. & HAKULINEN, T. (1978) Sunlight as a risk factor of malignant melanoma of the skin. *Cancer*, **41**, 2018.

VESSEY, M. P., DOLL, R., PETO, R., JOHNSON, B. & WIGGINS, P. (1976) A long-term follow-up study of women using different methods of contraception: An interim report. *J. Biosoc. Sci.*, **8**, 373.

WATERHOUSE, J., MUIR, C., CORREA, P. & POWELL, J. (1976) *Cancer Incidence in Five Continents*, Vol. III. Ed. Waterhouse *et al.* Lyon: IARC Scientific Publications.